# ***Tenebrio molitor*in the circular economy: a novel approach for plastic valorisation and PHA biological recovery**

**DOI:** 10.1007/s11356-021-15944-6

**Published:** 2021-08-28

**Authors:** Paola Sangiorgio, Alessandra Verardi, Salvatore Dimatteo, Anna Spagnoletta, Stefania Moliterni, Simona Errico

**Affiliations:** ENEA Italian National Agency for New Technologies, Energy and Sustainable Economic Development, Trisaia Research Centre, S.S. 106 Jonica, km 419,500, 75026 Rotondella, MT Italy

**Keywords:** Mealworm-based bioconversion, Food loss and waste, *Tenebrio molitor*, Novel food, PHA, Plastic biodegradation, Circular economy

## Abstract

The increase in the world population leads to rising demand and consumption of plastic raw materials; only a small percentage of plastics is recovered and recycled, increasing the quantity of waste released into the environment and losing its economic value. The plastics represent a great opportunity in the circular perspective of their reuse and recycling. Research is moving, on the one hand, to implement sustainable systems for plastic waste management and on the other to find new non-fossil-based plastics such as polyhydroxyalkanoates (PHAs). In this review, we focus our attention on *Tenebrio molitor* (TM) as a valuable solution for plastic biodegradation and biological recovery of new biopolymers (e.g. PHA) from plastic-producing microorganisms, exploiting its highly diversified gut microbiota. TM’s use for plastic pollution management is controversial. However, TM microbiota is recognised as a source of plastic-degrading microorganisms. TM-based plastic degradation is improved by co-feeding with food loss and waste as a dietary energy source, thus valorising these low-value substrates in a circular economy perspective. TM as a bioreactor is a valid alternative to traditional PHA recovery systems with the advantage of obtaining, in addition to highly pure PHA, protein biomass and rearing waste from which to produce fertilisers, chitin/chitosan, biochar and biodiesel. Finally, we describe the critical aspects of these TM-based approaches, mainly related to TM mass production, eventual food safety problems, possible release of microplastics and lack of dedicated legislation.

## Introduction

According to Plastics Europe (2020), in 2019, plastic production was 370 million tonnes worldwide and 58 million tonnes in Europe (Plastics Europe 2020). The growing demand and use of plastic are not yet accompanied by careful waste management, and very little is recycled (equal to 9% globally, as estimated by UNEP (2018)). Huge amounts of plastic materials are polluting even the most distant environments and turning oceans into “plastic soups”. In addition, microplastics are threateningly entering the food chain through marine animals. Each year, at least 8 million tons of plastic end up in the ocean, corresponding to the contents of a garbage truck thrown away every minute. If current trends continue, they are projected to increase to four per minute by 2050, so that for every 3 tonnes of fish, we will find 1 tonne of plastic in the ocean by 2025 and by 2050 more plastic than fish (by weight). In 2016, Ellen MacArthur Foundation (2016) estimated more than 150 million tonnes of plastic in the ocean, mostly made up of plastic packaging. Although plastics are a problem due to their disposal, they represent a great opportunity in the circular perspective of their reuse and recycling. Moreover, it is beyond doubt that they are an irreplaceable material in many sectors. For these reasons, research is moving not only towards sustainable systems for biodegrading plastic waste but also towards new non-fossil-based plastics that have similar characteristics and can be recycled, bio-based polymers such as PHAs.

**Fig. 1:**
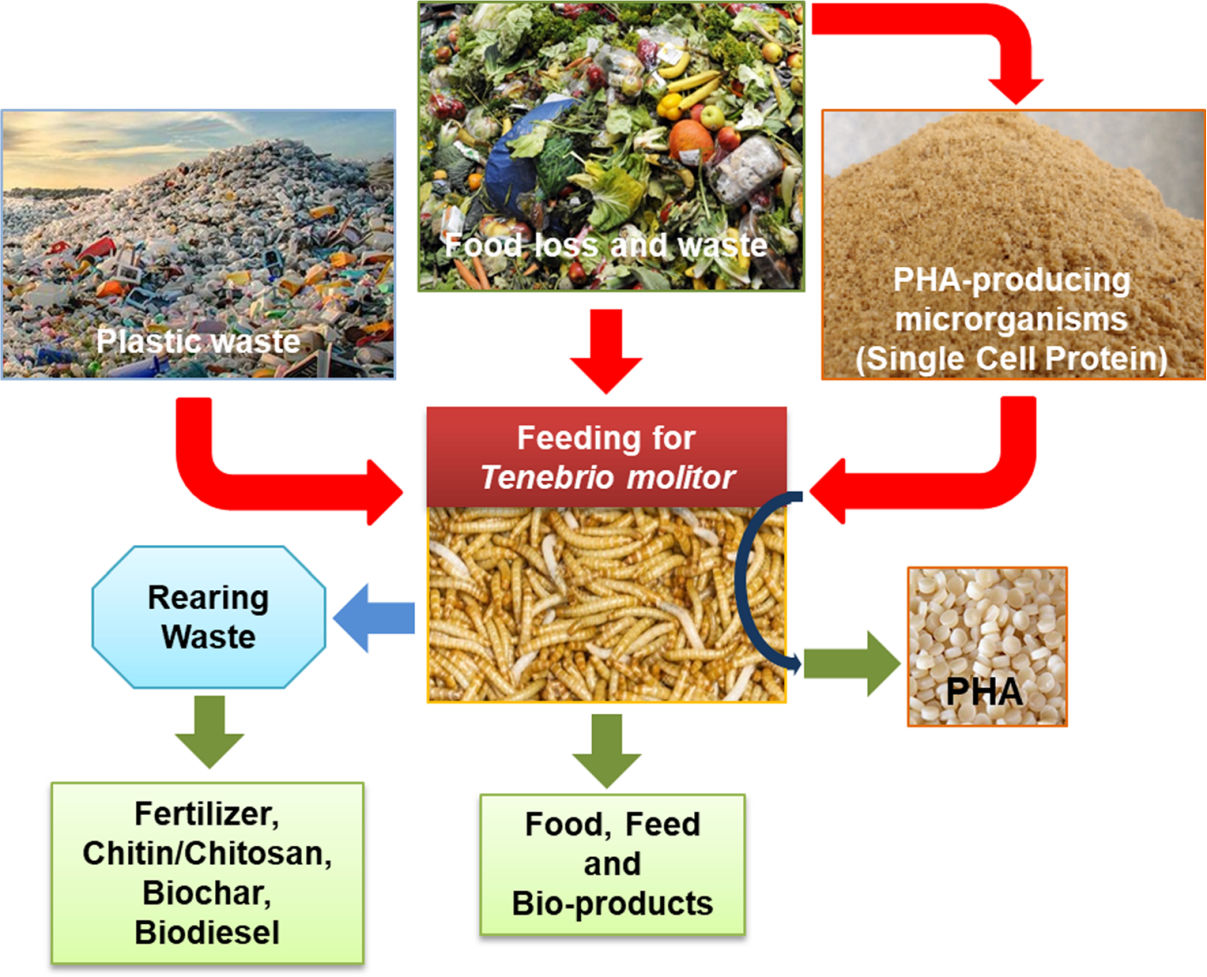
A novel approach of plastic valorisation and biological PHA recovery by using *Tenebrio molitor*

In this context, *Tenebrio molitor* (TM), an edible insect belonging to the family Tenebrionidae and Order Coleoptera, is gaining more and more attention in the scientific sector as a valuable solution for organic waste and plastic bioconversion, and biological PHA recovery, thanks to its highly diversified gut microbiota (Fig. 1).

TM is one of the few insect species able to decompose the lignocellulose matrix of resistant cellulose waste such as cardboard, and plastic waste, such as polystyrene (PS), polyethylene (PE), polypropylene (PP) and polyvinyl chloride (PVC) (Wu and Criddle 2021). TM-based plastic biodegradation is significantly enhanced by supplementing the TM diet with organic wastes; therefore, food loss and waste (FLW) could be used profitably. In this way, a twofold objective is achieved: the increase of the plastic degradation rate and the FLW valorization (Khan et al. 2021).

Currently, FLW represents approximately one-third of the global food production, amount to 1.3 billion tonnes per year of human food that has been lost or wasted (Ravi et al. 2020). It causes negative economic and environmental impacts, resulting in (i) an annual cost of over 1000 billion dollars; (ii) an output of around 3.3 billion tonnes of carbon dioxide equivalent per year and a carbon footprint that corresponds to about 7% of total anthropogenic GHG emissions; (iii) land consumption of almost 30% of the world agricultural area; and (iv) bluewater footprint of 250 km^3^ (6% of global water withdrawals) (FAO 2019).

**Fig. 2:**
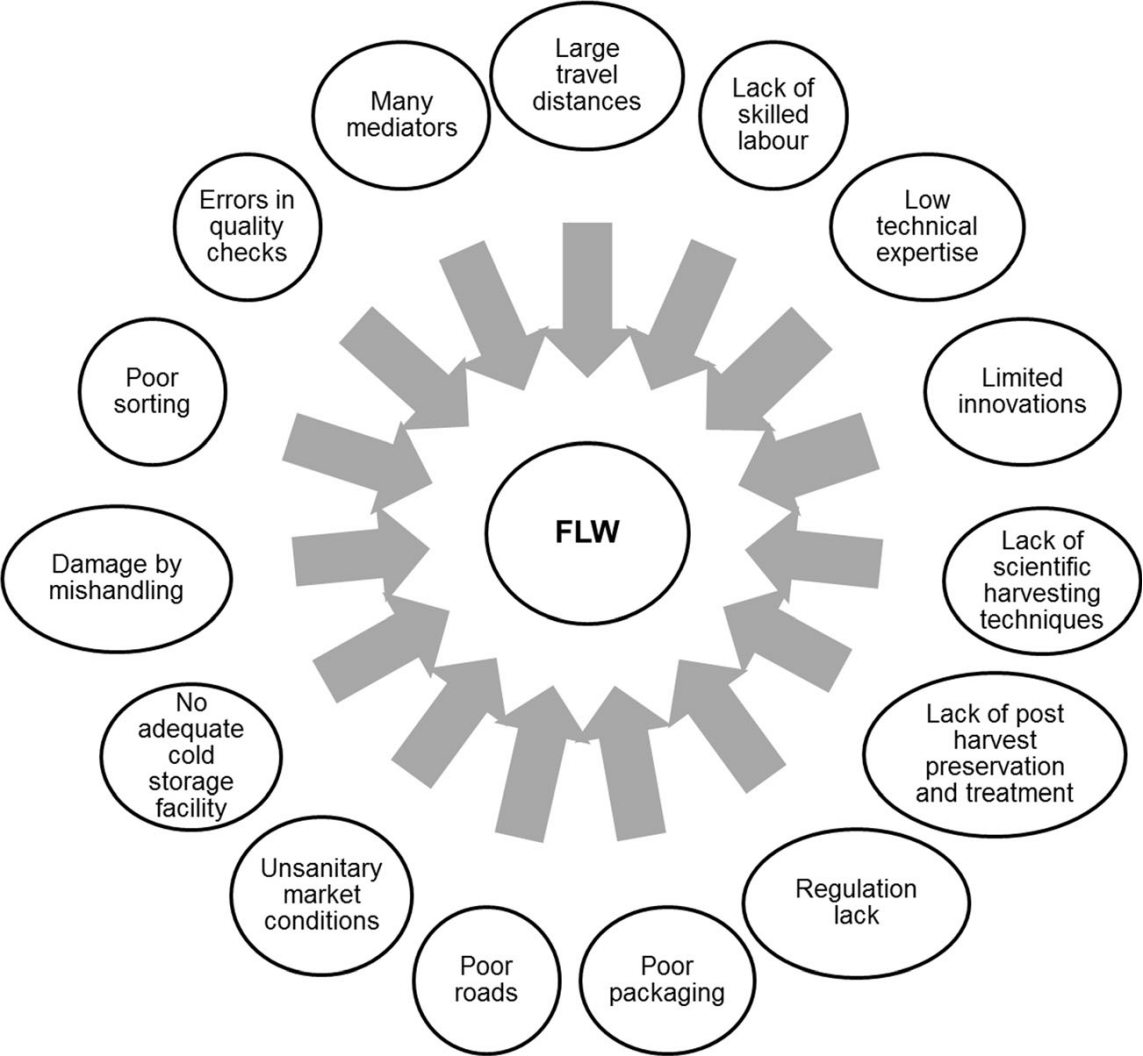
Factors leading to high FLW generation in food system chain

The high FLW levels are generated due to many factors throughout the entire food supply chain (FSC), as shown in Fig. 2 (Chauhan et al. 2021). To fight food waste with a circular view, FLW should be reinserted into the value chain, through a valorisation system, keeping its value as high as possible (Sangiorgio et al. 2020; Teigiserova et al. 2020) and providing economic opportunities and new job positions. The creation of a circular system must consider aspects related to the quality and seasonality of the FLW, the necessary logistics and the economic feasibility of the identified paths. At the same time, a close correlation must be created between all actors of the new value chains. In particular, since FLW is rich in nutrients and water, it is easily putrescible, with consequent odour problems and potential risk of mould, pathogens or toxins. Stabilisation processes such as homogenisation and pre-fermentation are required to preserve its quality and safety.

Pre-fermentation also helps improve the digestibility and bioavailability of nutrients for insects.

However, since insect rearing does not require specific geographical or natural environmental conditions—because it is conducted under controlled indoor conditions—it is suitable to be located close to the substrate suppliers, to minimise the handling of the feedstock and therefore costs (Mancini et al. 2019).

Additionally, TM and its gut microbiota are capable of releasing polyhydroxyalkanoates (PHAs) from plastic-producing microorganisms. PHAs are a group of natural biodegradable polyesters that can give bio-based biodegradable plastics, potentially replacing some petrochemical-based plastics. At present, one of the main drawbacks of PHA production is the high cost related to its recovery from the microorganism’s cell cytoplasm (Li et al. 2016). Organic toxic solvents, such as chloroform, acetone and methylene chloride, are mainly used for PHA extraction with negative environmental and safety consequences (Ong et al. 2018b). A valid alternative system for PHA recovery uses TM larvae as an eco-friendly and cost-effective downstream strategy that provides high purity PHAs (Haddadi et al. 2019). A further advantage of this biological PHA recovery is the possibility of feeding plastic-producing microorganisms with FLW. In this way, it is possible to increase the cost-effectiveness of the entire process and promote a circular economy (CE) perspective, where the “end-of-life” concept is replaced by the 3Rs strategy “Reduce, Reuse, Recycle”. Following the CE approach, materials can be recovered in all phases of FSC (Kirchherr et al. 2017), providing financial gains from what is otherwise useless or a cost.

TM is an alternative source of protein and micronutrients, showing a low ecological footprint compared to traditional livestock. TM larvae produce lower GHG and need much less land and water than other meat animals (Oonincx and de Boer 2012; Yang and Wu 2020). For these reasons, TM is among the insect species authorised by the European Union (EU) in aquaculture and feeding pets (European Commission Regulation 2017/893). TM authorization for ruminants (e.g. sheep and cattle) and monogastric animals (e.g. pigs and poultry) is expected by the end of 2021.

In addition, TM is the first insect approved as a novel food by EU (Commission Implementing Regulation 2021/882), after the favourable evaluation of the European Food Safety Authority (EFSA) concerning the potential risks associated with TM consumption, such as the presence and accumulation of parasites, microorganisms, mycotoxins and heavy metals (EFSA 2021).

However, the existing legislation on the insects’ use as feed and food differs widely between countries worldwide (Sogari et al. 2019).

Despite its potential value, TM mass production is not yet environmentally and economically sustainable. Scaling up requires large investments to fund staff increases and automated facilities. In addition, large-scale production should find alternative substrate sources to replace expensive and unsustainable commercial feeds. In this context, the use of FLW as feed for TM could be a viable solution, provided that it complies with legislative and food safety constraints and there is no competition with other animals (FAO 2021).

Furthermore, it is crucial to improve the transformation processes of the TM larvae into the final products—such as flours, oils and protein extracts—in terms of food safety, cost-effectiveness and environmental sustainability (Melgar-Lalanne et al. 2019).

In this review, we examine the most recent articles on the use of TM as a possible solution to the environmental and socio-economic problems related to the scenario described so far. We focus on the ability of TM, together with its gut microbiota, to biodegrade plastic waste, and its strong potential in the biological recovery of PHA, in the perspective of the circular economy.

Finally, we highlight critical aspects related to the use of PS-degrading or PHA-producing TM for human or animal consumption, the release of microplastics and other contaminants and the lack of dedicated legislation.

## Biodegradation of plastic waste

New approaches to plastic degradation using insect caterpillars and their gut microbiota have opened up new ways to solve the plastic environmental problem (Yang and Wu 2020; Khan et al. 2021).

Recent studies have shown that others insects in addition to TM appear to contribute to and accelerate the biodegradation rate of some recalcitrant plastics (Table 1).

**Table 1:** Some insects able to degrade some recalcitrant plastics

**Tested insect**	**Degraded polymer type**	**Ref.**
*Plodia interpunctella* larvae	PE	Yang et al. (2015a)
*Galleria mellonella* larvae	PE	Bombelli et al. (2017) Ren et al. (2019) Cassone et al. (2020)
Dark mealworms (*Tenebrio obscures*)	PS	Peng et al. (2019)
Superworms (*Zophobas atratus*)	PE and PS	Peng et al. (2020a)
Lesser waxworms (*Achroia Grisella*)	HDPE	Kundungal et al. (2019)
Land snails (*Achatina fulica*)	PS	Song et al. (2020)

*PS* polystyrene, *PE* polyethylene, *HDPE* high-density PE

Table 2 shows an overview of the main results of the degradation of plastic polymers by TM in different test periods and for several plastic materials; consumption rates were converted for consistency into milligrammes of polymer consumed per 100 larvae per day.

**Table 2:** Consumption rates of different plastic polymers by *Tenebrio molitor*

**Polymer type**	**Sample for study**	**Test duration (day)**	**Consumption (mg)/100 larvae/day**	**Ref.**
PE	LDPE foam	32	23.04	Brandon et al. (2018)
PE	LDPE film	60	3.45	Yang et al. (2021)
PE (PE PS mix)	LDPE foam + EPS Foam	32	16	Yang et al. (2021)
PE + half WB	LDPE foam	32	32	
PE	Commercial fruit bag LDPE	38	2.3	Billen et al. (2020)
PE	LDPE foam	60	3.33	Yang et al. (2021)
PE	Loosely folded cling film LDPE	38	0.5	Billen et al. (2020)
PE micro	Micro LDPE	28	18.42, 10.99, 14.84*	Wu et al. (2019)
PLA	In the form of 1-mm-thick plates	21	15	Bozek et al. (2017)
PP	PP foam	35	1	Yang et al. (2020)
PP + half WB	PP foam	35	1.6	
PS	EPS foam	32	22.2, 16.9**	Yang et al. (2018a)
PS (PE PS mix)	LDPE foam + EPS Foam	32	7.04	Brandon et al. (2018)
PS	XPS coffee cup	32	10.2	Yang et al. (2018a)
PS	XPS food packaging containers	32	14.4–17.0	
PS + half WB	EPS foam	32	27.04	Brandon et al. (2018)
PS + soy protein	EPS foam	32	49.1	Yang et al. (2018a)
PS + WB	EPS foam	32	44.1	
PS + WB (16:1) a 25°C	EPS foam	32	84	
PS + WB (16:1) a 30°C	EPS foam	35	78.5	
PS + WB (8:1) a 20°C	EPS foam	32	67.6	
PS	EPS foam	32	15.04	Brandon et al. (2018)
PS	EPS foam	31	24.3	Peng et al. (2020b)
PS	Material for parcels	21	23.8	Urbanek et al. (2020)
PS	XPS in blocks	30	8.8	Yang et al. (2015c)
PS	PS foam	60	4.27	Yang et al. (2021)
PS micro	Micro PS	28	28.70, 17.19, 25.69*	Wu et al. (2019)
PS	In the form of 1-mm-thick plates	21	25.48	Bozek et al. (2017)
PS	In the form of 1-mm-thick plates	21	6.34	Urbanek et al. (2020)
PS	In the form of powder	21	12.69	
PS	EPS	21	28.57	
PVC micro	Micro PVC	16	36.6	Peng et al. (2020b)
PVC micro	Micro PVC	28	24.19, 16.99, 28.49*	Wu et al. (2019)
PVC	Tubing for oxygen supply, cut in 10mm pieces	21	13.57	Bozek et al. (2017)
Tire crumb	In powder form	21	3.4	Aboelkheir et al. (2019)
v-SBR	In powder form	21	6	

Data from literature, expressed as mg of plastic matrix consumed per 100 larvae per day. *PS* polystyrene, *PE* polyethylene, *LDPE* low-density PE, *PP* polypropylene, *PVC* polyvinyl chloride, *PLA* polylactic acid, *v-SBR* vulcanised butadiene-styrene elastomer, *micro* microparticulate, *EPS* expanded polystyrene, *XPS* extruded polystyrene, *WB* wheat bran

*Results using TM from 3 different Chinese regions

**Second generation juvenile larvae weighing 30 mg

As first shown by Yang et al. (2015c), biodegradation and mineralisation of polystyrene (PS) occur in the gut of mealworms; TM can be compared to an efficient bioreactor degrading PS in a multi-stage process and leading to the breakdown of long-chain PS molecules into low molecular weight metabolites. TM larvae start the biodegradation process by chewing PS, thus promoting contact between the increased PS surface and microorganisms/extracellular enzymes in the larval digestive tract. When the crushed PS reaches the gut, it meets TM gut microbiota, which secretes enzymes that catalyse the PS decomposition into fragments. The entire process is based on the synergy between microorganisms and their mealworm host. PS was efficiently degraded and egested in less than 24 h. PS carbon was converted up to 47.7% into CO_2_ in 16 days, with only a limited fraction (ca. 0.5%) incorporated into fatty acids; however, PS-fed larvae lost 24.9% of their biomass. No significant differences in survival rate were observed between TM larvae fed on PS as the sole diet and those fed on a standard diet (bran) over 1 month. Subsequent studies, Yang et al. (2015b) found that suppression of gut microbiota using proper antibiotics (gentamicin) compromised the TM ability for PS biodegradation and mineralisation. Moreover, they isolated from the plastic-eating larvae gut a PS-degrading bacterial strain, *Exiguobacterium* sp. strain YT2, capable of degrading 7.4% of the PS pieces over a 60-day incubation period. This PS degradation efficiency is lower than that obtained by TM in Yang et al. (2015c) (up to 47.7%); this is probably due to the lack of the above-mentioned synergy between microbiota and host.

A global collective work has verified the TM degradation capacity of PS; TM eats and metabolises PS regardless of geographic origin (Yang et al. 2018b). Wu et al. (2019) confirmed this result in the case of both PS and low-density polyethylene (LDPE) degradation by TM, but not in the case of polyvinyl chloride (PVC) metabolisation; the authors speculated that PVC is less easily digested by mealworms.

Yang et al. (2021) confirmed the role of the gut microbiota in degrading PS by antibiotic suppression tests. However, in the LDPE case, gentamicin inhibits LDPE depolymerisation only partially. Here, the authors observed less gut microbe dependence. The analysis of the gut microbial community indicated that the microbiome significantly differs in its composition depending on different diets (bran, PS and LDPE), likely due to the development of microbes associated with the different substrates.

Among the various plastic materials, matrices extremely resistant to biodegradation, such as vulcanised styrene-butadiene rubber (v-SBR) and tire crumb, have also been studied (Aboelkheir et al. 2019). After 3 weeks of direct contact as their only meal, TM larvae biodegrade the vulcanised rubber polymeric chains, causing a bio-desulfurization accompanied by a rubber degradation. Despite that, the consumption rates are very low compared to those obtained for other plastic materials. The survival rate was 100%; however, larvae fed on tire crumb had a weight loss of 10.54%, whereas larvae fed on v-SBR and bran showed a mass gain of 9.31% and 22.23%, respectively.

Peng et al. (2020b) tested rigid PVC microplastic powders as the sole diet of mealworms. They found broad depolymerisation witnessed by the reduction of weight, number and size average values of the polymer (33.4%, 32.8% and 36.4%, respectively). The egested frass contained about 34.6% of residual PVC polymer, together with a small fraction of chloride (only about 2.9% of the PVC ingested). These results indicate good depolymerisation but limited mineralisation of the PVC. They also found a gut microbiome dependence of PVC depolymerisation. The authors concluded that the use of TM for PVC bioremediation is not a reliable approach. However, they stated that it is necessary to understand better both the biodegradation pathways and the synergistic interactions between plastic-degrading microorganisms and TM digestive system.

As all plastics contain various types of additives to improve the properties of polymers, environmental pollution problems can also arise due to these contaminants. Brandon et al. (2020) investigated the fate of hexabromocyclododecane (HBCD), the most common flame retardant for PS, in PS-degrading larvae and, further up the food chain, in TM-fed shrimp. They did not observe bioaccumulation or toxicity. However, HBCD is just one of many plastic additives. Further studies are therefore needed to assess the fate of other plastic-derived contaminants in the food chain (Brandon et al. 2020).

In addition, it is necessary the effect of residual plastic particles, which are smaller and more contaminated in chemical substances and therefore can cause nanoscale toxicity (Jiang et al. 2020).

For this reason, researchers have recently studied the substances generated in TM biomass during insect degradation of PS. In a 7-day test, they identified several oligomers resulting from PS biodegradation, such as PS monomers (e.g. styrene, α-methyl styrene, acetophenone), oligomers and other unintentionally added substances (Tsochatzis et al. 2021). For all residual substances (except α-methyl styrene which reaches a plateau up to day 7), after an initial increase from day 1 to day 3, a decrease is observed on day 7. This trend can be explained by the enzymatic conversion of ingested compounds into CO_2_, as already reported by Yang et al. (2015c). In parallel, Tsochatzis et al. (2021) identified several bioactive components, such as myristic, palmitic and oleic acids and their respective amides. The authors concluded that the absence of hazardous chemicals in TM biomass and the presence of bioactive molecules suggest that plastic biodegradation by TM appears to be a valid bio-recycling process. Several authors have observed mass losses in the mealworms fed for a long time with plastic material as the only feed source. Weight losses were sometimes similar to those of starved larvae: they suggested that plastic degradation can, at best, provide enough energy for survival but cannot provide all needed nutrients (Wu et al. 2019; Urbanek et al. 2020). The addition of feed as soy or WB can support TM larvae to grow and complete their life cycle (Peng et al. 2020b; Urbanek et al. 2020; Yang et al. 2020). Moreover, proper amounts of extra feed can enhance plastic degradation (Brandon et al. 2018; Wu et al. 2019).

Yang et al. (2018a) mixed PS with bran and observed that mealworms eat first bran, then PS. PS eaten percentage is higher than that of larvae fed with PS alone; consumption rates generally increase with increasing WB:PS ratios, but the higher the ratios, the more the larvae prefer the bran and the lower the PS degradation. At optimal WB:PS ratios, TM complete all life cycle stages (larvae, pupae, beetles, egg); the second generation juvenile larvae (about 30 mg) have consumption rates in the range of values obtained for the mature first-generation mealworms (about 80mg), showing that it is possible to proceed with selective breeding (Yang et al. 2018a). In a similar 35-day study, Yang et al. (2020) confirmed the positive effect of WB addition on TM degradation and PP consumption rate; the presence of WB decreased cannibalism and slightly increased survival rate. However, unfed larvae showed a much higher cannibal rate (31.6%) and lower survival (68.4%), indicating that polypropylene (PP) somehow supports TM development.

Matyja et al. (2020) used the dynamic energy budget (DEB) model to analyse the effects of PS (as a single diet or added to oat) on the growth and metabolism of TM in a 91-day test. They observed not only losses in both TM mass and PS samples, as also reported by Yang et al. (2015c and 2018a), but an enhancement of PS consumption rates when oat is added to PS. However, they concluded that changes in the development of PS fed larvae are due to a decrease of reserves and physiological reaction to insufficient food intake. Mealworms can face the condition of starvation and become pupae, not thanks to the ingested PS but the energy provided by their reserves.

A recent work by Peng et al. (2021) achieved a conversion efficiency of 81.5–86.9% with mixtures of polylactic acid (PLA) and bran and an optimal yield of TM biomass at a ratio of 20% PLA. The authors propose a circular approach for the management of PLA waste. PLA is used as feed for the production of TM larvae (for food and feed); mealworm droppings are used as fertiliser to have corn crops (for food, feed and industrial applications), and related agricultural by-products are used in the production of PLA that re-enters the cycle.

The possibility of using TM for the bioremediation of plastics has received not only enthusiastic evaluations but also some critics. Among the critical researchers, Billen et al. (2020) studied polyethylene (PE) and PS biodegradation by macro-organisms such as TM and wax moth larvae. They tested both live specimens and homogenate paste. Their experiments show that larvae apparently chew and digest the plastic, changing the morphology of the substrate. Furthermore, they observed a plasticizing action by an excreted liquid. This evidence indicates that the degradation mechanism involves more than just the gut microbiome. They also observed that the ability to chew, ingest and biodegrade plastic is too low to handle the huge volume of plastic waste. Since the larvae appear to survive with PE and not grow, mealworms must be reared before being employed in PE degradation, with an increase in process costs, as discussed in paragraph 4. Even the lipid fraction, a biodiesel precursor, does not derive from the plastic conversion but from other parts of their diet. The authors argue that only if glycol-like fractions or other building blocks (of higher value than biodiesel) could result from biodegradation, the outcomes of their techno-economic analysis would change. Moreover, the non-digested microplastics observed in residues of TM fed on plastic substrates are a risk to public health and the environment. Therefore, this rearing waste can only be incinerated with energy recovery.

However, Billen et al. (2020) admitted the importance of understanding the plastic degradation mechanism to develop new green methods, such as the inoculation of food waste with plastic-degrading microorganisms to mitigate plastic anomalies during composting or digesting, or the functionalization of alkane moieties.

The article by Khan et al. (2021) disputes the conclusions of Billen et al. (2020) on the uneconomic nature of TM plastic degradation. Khan et al. (2021) state that, in their analyses, they used eerily much lower degradation rates than those found in the literature and did not consider high value-added insect-based compounds such as proteins. However, they agree that, for effective exploitation of plastic waste, insects must convert these substrates quickly and into biomass or useful by-products; furthermore, in order for the process not to lead to environmental and health problems, neither the biomass nor the residue must contain microplastics or harmful additives.

Parallel to the study of the biodegradation of plastic materials through the use of insects, research is increasingly moving towards the study of bacterial strains isolated from plastic-degrading larvae and their ability to degrade plastic on their own. The aforementioned isolation of the bacterial strain *Exiguobacterium* sp. capable of degrading PS (Khan et al. 2021) is the first research in this context.

Urbanek et al. (2020) isolated from TM gut three bacterial strains belonging to species of *Serratia marcescens*, *Klebsiella oxytoca* and *Pseudomonas aeruginosa*. They tested them on bioplastics such as polybutylene succinate adipate (PBSA), polybutylene succinate (PBS) and polycaprolactone (PCL) and found a good biodegradation ability.

Yin et al. (2020) studied the combination of two bacterial isolates from TM guts (*Acinetobacter* sp. strain NyZ450 and *Bacillus* sp. strain NyZ451) for the biodegradation of PE mulching films. This bacterial consortium could grow on PE as the sole carbon source, removing 18% of PE film after 30 days, even though each strain had no such ability on its own. The synergy between these two strains seems to enhance the capability of activating recalcitrant substrate via complementary catabolic pathways.

However, Khan et al. (2021) suggest expanding the study to fungi derived from the insect gut because, unlike bacterial enzymes, little has been studied about fungal enzymes. Other ways to explore in the future could be (i) the study of gut microbiota of insects not optimally reared but fed with mixed wastes (organic and plastic wastes as normally found) and (ii) the inoculation of plastic-degrading microorganisms (bacteria and fungi) into insect species.

In conclusion, based on the literature and on our direct experience (data not yet published), the use of insects cannot realistically be considered a valid remediation strategy for plastic pollution. However, the study of the richness of their microbiota—function of the type of plastic with which the insect comes into contact—can be used to identify strategic microorganisms for plastic mitigation processes.

## Biological recovery of PHA using mealworms

Polyhydroxyalkanoates (PHAs) are a group of natural biodegradable polyesters synthesised by several microorganisms such as *Aeromonas*, *Azotobacter*, *Cupriavidus*, *Clostridium*, *Methylobacterium*, *Ralstonia*, *Pseudomonas* and *Syntrophomonas* (Khan et al. 2021). PHAs are produced by these prokaryotes when key non-carbonaceous nutrients (e.g. nitrogen or phosphorus) are limiting. In this way, PHA accumulation as energy storage improves survival of microorganisms under stress conditions.

PHAs are potentially able to replace some petrochemical-based plastics due to their high biocompatibility and biodegradability in different environments and the release of non-polluting and non-toxic products after degradation (Bhatia et al. 2019).

The global PHA market is estimated at 215.2 million USD in 2020, and it is expected to reach 327.3 million USD by the end of 2026, with a CAGR of 6.1% over the forecast period 2021–2026 (360ResearchReports 2020). This review analyses the knowledge about the use of TM as biological recovery tools in PHA production.

Despite the great potentiality, some disadvantages limit PHAs’ competition with traditional synthetic plastics or their application as ideal biomaterials. Among these, one of the main limitations is their high production cost (Li et al. 2016), specifically for the PHA recovery process. Since PHAs are accumulated in the bacterial cell cytoplasm, it is necessary to lyse cells to recover the PHA granules. Some of the PHA recovery methods include the use of solvents (chloroform, acetone, methylene chloride), enzymatic and hypochlorite digestion. The extraction with organic solvents is the most usual and best than the others but involves environmental and safety consequences (Ong et al. 2018a).

An alternative PHA recovery system foresees the use of small animals fed with dried bacterial cells containing PHA to let them digest the cells and defecate the released PHA (Murugan et al. 2016).

Kunasundari et al. (2013) had previously used the Sprague Dawley rats to purify P(3HB), biopolymers belonging to the PHAs family. They managed to extract almost 90 wt% of PHA from white faecal pellets. A similar approach has been carried out by Murugan et al. (2016), who used TM as the animal model to purify P(3HB-co-3HHx), a PHA co-polyester.

The insects, such as cricket, cockroach, superworm and mealworm, are much easier to maintain. They require minimal resources and space and can efficiently produce frass with recoverable purified PHA. Although not yet perfect, this process provides a new option for the recovery of PHA where highly pure PHA is unnecessary. At present, the biological recovery of PHA with TM larvae is considered one of the best available techniques (Ong et al. 2018b).

Comparison of cell disruption methods needed to free PHA from bacterial storage reveals that the usual extraction ones (i.e. virus lysis) have more concerns than using rats and mealworms (Kourmentza et al. 2017). Compared to bacteriophage-mediated lysis, the TM digestion system is considered an ecological and cost-effective downstream strategy and provides high purity polyhydroxybutyrate (PHB) (89%) (Haddadi et al. 2019).

It is necessary to consider that, for the complete biological PHA recovery, the microbial cells have to be harvested and pre-treated and, after animal digestion, accumulated, polished and dried. Among all these phases, the drying technique, which usually consists of heat drying or lyophilisation, has heavy economic and technical issues (Zainab-L and Sudesh 2019).

In Ong et al. (2018a), PHA granules obtained from the biological recovery with TM were not only close to those produced with conventional recovery systems but also similar to those in the bacterial cells. As revealed by TEM and SEM micrographs, the PHA granules remained spherical, with no morphological changes. The study also demonstrated that bacterial cells were readily consumed by TM larvae. Moreover, 16S rRNA metagenomic sequencing showed a correlation between the feed type and the gut microbiome (Ong et al. 2018a).

As also shown by Murugan et al. (2016), the ability of TM to excrete PHA as granules after consumption of freeze-dried cells of *Cupriavidus necator* containing PHA granules is the best-tested pathway for polymer recovering with small animals. Mealworms indeed digest cells but not PHA granules. Subsequent purification of PHA granules with water, detergent and heat was sufficient to give pure PHA with no reduction of the molecular weight and no dispersion of molecules, with the same results compared to conventional chloroform extraction. The morphology of PHA recovered from TM digestion, analysed by electron microscopy and dynamic light scattering measurements, was also kept unaltered.

Additional interest can derive from the utilisation of this biorefinery strategy for the valorisation of agri-food by-products. According to this approach, microorganisms can be fed on waste and by-products from the food and agricultural industries, produce PHA, and then, once freeze-dried, be used as feed for mealworms that release PHA from the microorganism cells (Zainab-L and Sudesh 2019; 360ResearchReports 2020). As reported by some authors, a lot of waste plant and animal feedstock can be used as a source of carbon for a variety of microorganisms, each producing different types of PHA. These by-products include used cooking oils, plant oils with no value for human consumption, fish oils and dairy processing waste (Surendran et al. 2020; Dutt Tripathi et al. 2021; Kalia et al. 2021).

**Fig. 3:**
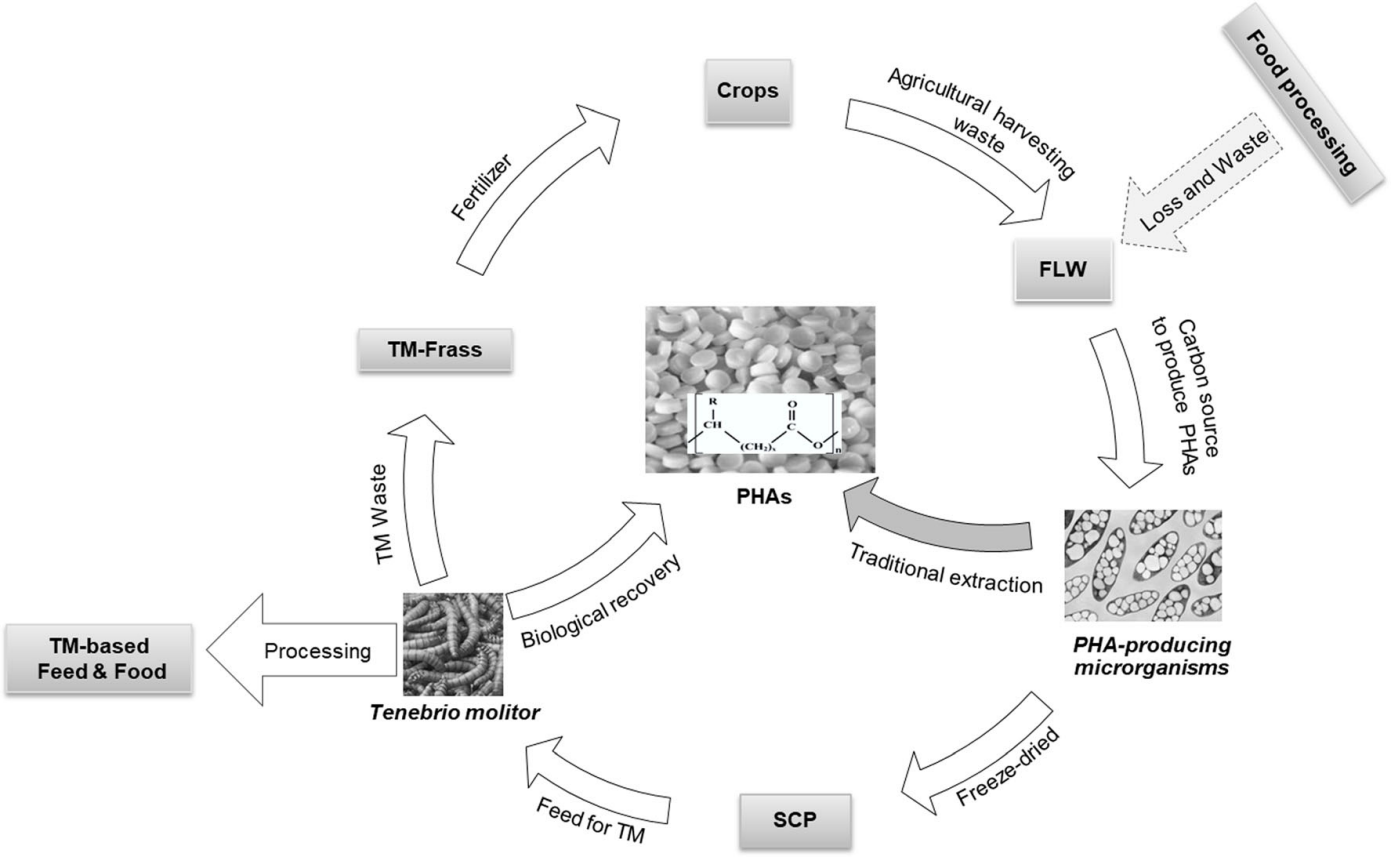
*Tenebrio molitor* in a circular strategy for the biological recovery of PHA, the production of feed and food and the valorisation of agri-food waste. Abbreviations: C carbon, FLW food loss and waste, PHAs polyhydroxyalkanoates, SCP single-cell protein, TM *Tenebrio molitor* (Chee et al. 2019)

As noted by Chee et al. (2019), the microorganism capable of producing PHA, such as *Cupriavidus necator*, can be used both as a single-cell protein for feed and food and as a source from which PHA granules can be recovered simultaneously. Thus, by feeding and digesting these single-cell proteins, TM releases PHA for plastic packaging and provides animal biomass for food and feed, and frass as fertiliser to be used for the growth of new crops. In addition, by-products derived from these crops, together with other food processing wastes, can be the source of carbon to grow *Cupriavidus* that feeds TM and so on (Fig.3).

Following this approach, PHA was produced by using *Pseudomonas mendocina* cultured for 72 h in mineral salt medium containing biodiesel liquid waste (2% v/v) and then recovered and purified using mealworms. Released PHA had high purity and higher molecular weight than that recovered using chloroform extraction, thus confirming the feasibility of the process and the ability of TM to extract PHA from various types of bacterial cells (Chee et al. 2019). In addition to the valorisation of waste and by-products by closing the PHA production cycle, research is trying to improve the efficiency of the recovery system by TM in order to reduce its production cost. One way to do that is the improvement of the consumption of PHA-containing cells by TM. Zainab-L and Sudesh (2019) proposed a simple washing method to reduce the level of mineral salts (deriving from the culture medium) in lyophilised cells to increase their palatability for TM and, consequently, the quantity of PHA in TM faecal pellets. In this way, the authors demonstrated a significant improvement in the production, recovery and purity of PHA. At the same time, in the resulting larvae, they observed an increase in the protein fraction (79%) and a reduction in the fat content (8.3%).

## Critical aspects of plastic biodegradation and PHA recovery through TM

One of the chief limits to the use of TM for plastic biodegradation or PHA recovery processes is related to the high cost of insect mass production to obtain the larvae.

Billen et al. (2020) observed the poor ability of TM to chew, ingest and biodegrade a large volume of plastic waste. They argue that mealworms’ rearing proves necessary before employing larvae in plastic degradation.

For instance, assuming a theoretically maximum complete degradation rate of approximately 0.22 mg PE per worm per day, up to 10 tonnes of larvae would be required to treat 1 tonne of PE. From a technical-economic point of view, this system is not feasible due to the slowness and cost of TM rearing compared to the larvae effect on plastic waste management.

TM production results in less GHG emissions and requires much less land than chickens, pigs and cattle (Oonincx and de Boer 2012).

However, when Le Féon et al. (2019) compared the environmental impacts of 1 kg of trout obtained by replacing fishmeal with different levels of TM, they found an increase in energy demand and land use as the percentage of added TM increased. On the other hand, water consumption and use of biotic resources were lower.

Moreover, the scaling up of TM rearing is currently more expensive and then uncompetitive compared with traditional livestock or farming sources. Edible insect rearing requires multiple investments to build facilities, develop automation of both rearing and processing methods and recruit more skilled staff, with an overall increase in production costs (Maillard et al. 2018; FAO 2021). Furthermore, the inadequacy of sound scientific knowledge relating to methods and technologies, dietary formulas and control conditions for the rearing of insect’s huge volumes limits the transition to industrial-level TM production (van Huis 2013; Cadinu et al. 2020).

Thus, insects’ production across Europe is largely concentrated in household and small-scale operations, and only a few large-scale companies in the sector are operating.

For this reason, the EU and the Bio-based Industries Joint Undertaking helped fund projects such as “Farmyng” (2019–2023) to develop innovative supply chains that include industrial insect rearing and marketing insect-derived products, thus attracting the interest of major investors.

The sustainability of the TM mass production process can be improved by using FLW to replace commercial feed. TM can be grown on low-value organic material and transform into high-quality food or feed.

The removal of FLW from poor environmental management has positive impacts on climate change and livelihoods and allows for the conservation of natural resources used for FLW production (van Huis and Oonincx 2017; Cadinu et al. 2020). It results in economic benefits both to producers involved in harvest and post-harvest operations who suffer FLW-related income losses and consumers who undergo FLW’s contribution to tightening the food market and rising food prices (Wunderlich and Martinez 2018).

In particular, fruits and vegetable wastes are ideal substrates to rear edible insects (Ravi et al. 2020). However, according to the Commission Regulation (EU) No 68/2013 on the Catalogue of feed materials, insects cannot feed on foodstuffs contaminated with pathogenic agents, as well as animal by-products or catering wastes. Furthermore, the potential contamination of waste with heavy metals, mycotoxins, pesticides or other hazardous materials must be considered (Fowles and Nansen 2020).

Currently, little information is available concerning optimal diets for TM growth at a large scale. TM biomass conversion into a wide range of products of commercial interest, such as feed and food, chitin, chitosan, biomaterials, fertilisers and biofuels, can help ensure the sustainability of the production process and therefore the transition towards a circular economy model (Azagoh et al. 2015; Ojha et al. 2020).

A large part of the population of Western countries is currently reluctant to eat edible insects due to psychological and cultural barriers. For this reason, it is preferred to incorporate insects into food as ingredients (such as flour, oils and protein extracts) to remove them from the consumer’s sight (Guiné et al. 2021). The manufacture of insect-based products must preserve their nutritional quality, food safety and shelf life. The optimization of transformation processes can increase the environmental and economic sustainability of TM-based products (Melgar-Lalanne et al. 2019).

Although scientific interest in TM larvae is mainly due to their nutritional potential, the possibility to use PS-degrading or PHA-producing TM for human or animal consumption has not yet been explored. Furthermore, there are currently regulatory gaps in these fields. Some scientific work on TM larvae fed with different diets supplemented with PS waste suggested the absence of hazardous chemicals in TM biomass accompanied by the presence of bioactive molecules (Brandon et al. 2020; Tsochatzis et al. 2021). Then, including this novel nutrition form could enhance TM value. However, it should be noted that plastic degradation by TM could generate microplastics due to incomplete degradation and mineralisation. Microplastics harm the environment and human health since they attract and store toxic compounds, such as polychlorinated biphenyls (PCBs), polybrominated diphenyl ethers (PBDs) and bisphenols (Mohanan et al. 2020). Furthermore, all plastics contain various types of contaminants. Currently, only the HBCD additive was investigated by Brandon et al. (2020) in PS-degrading larvae and further up the food chain in TM-fed shrimp. Then, further studies are needed to evaluate the presence of several plastic-derived substances able to cause different toxic effects (Jiang et al. 2020). Consequently, lots of fundamental research remains to be done, also to fill current regulatory gaps.

## Conclusion

The academia and the business world have proved TM to be a strategic insect from a circular economy point of view and a bioeconomy perspective. Thanks to its highly diversified gut microbiota, TM can grow on low-value substrates. In this way, its biomass provides food, while its rearing waste gives fertilisers and bioproducts such as chitin, chitosan, biochar and biodiesel. Furthermore, TM can bioconvert plastic waste and release PHA from plastic-producing microorganisms, acting as an effective bioreactor.

This review is a critical look at the existing research on plastic degradation and PHA biological recovery by TM. The use of mealworms to manage plastic pollution is still an open debate: to degrade the enormous volume of plastic waste, it would be necessary to have an equally huge amount of larvae that must be co-fed to promote the rate of degradation. However, most researchers acknowledge that the TM microbiota may be a source of microorganisms useful for plastic biodegradation. Furthermore, the possibility of using FLW as a co-feeding can make the process more advantageous, pursuing two objectives: on the one hand, the valorisation of waste of no value (or even a cost) and on the other the degradation of plastic waste. The biological recovery of PHA from PHA-producing microorganisms using TM as a bioreactor is a valid alternative to traditional lysis systems of the cell walls and extraction of the polymer. Here too, it is possible to make the process more advantageous in economic and environmental terms: FLW feeds the microorganisms that, in turn, will supply feed to TM in the form of single-cell protein. TM, together with the release of PHA, provides protein biomass and valuable rearing waste.

Much remains to be done, especially for the development of optimal conditions of TM mass production from an environmental and socio-economic point of view. At the same time, in-depth studies will be needed to uncover the potential of TM’s gut microbiota to facilitate the management of huge volumes of plastics.

The recent favourable opinion of EFSA (2021) and the even more recent preliminary approval by the European Commission (2021) for the use of TM as a novel food represent a push towards the production of TM as an alternative protein source. This push will determine a greater knowledge of the full potential of TM and its gut microbiota, to use this insect also as a tool to advantageously convert FLW and plastic materials and as a bioreactor for PHA recovery.

## Data Availability

The authors can confirm that all relevant data are included in the article, mentioned in the reference list.
